# Chest Wall Reconstruction Using a Titanium Mesh Plate and an Expanded Polytetrafluoroethylene Sheet: A Case Report

**DOI:** 10.70352/scrj.cr.25-0381

**Published:** 2025-10-11

**Authors:** Takahide Toyoda, Taichi Suzuki, Yuki Hirai, Ryo Karita, Taisuke Kaiho, Kazuhisa Tanaka, Yuichi Sakairi, Hajime Tamura, Ichiro Yoshino, Hidemi Suzuki

**Affiliations:** 1 Department of General Thoracic Surgery, Chiba University Graduate School of Medicine, Chiba, Chiba, Japan; 2 School of Medicine, Chiba University, Chiba, Chiba, Japan; 3 Department of Thoracic Surgery, International University of Health and Welfare Narita Hospital, Narita, Chiba, Japan

**Keywords:** chest wall tumor, chest wall reconstruction, rigid reconstruction, titanium plate, extended polytetrafluoroethylene sheet, case report

## Abstract

**INTRODUCTION:**

The integrity and stability of the chest wall (CW) are major factors that ensure the protection of the thoracic organs and proper respiratory function. In cases requiring extensive CW resection for tumor control, reconstruction using autologous tissue or synthetic materials is often performed. However, the optimal approach remains undetermined. We report a case of successful CW reconstruction using a combination of a titanium mesh plate and a dual-surface expanded polytetrafluoroethylene (ePTFE) sheet.

**CASE PRESENTATION:**

A man in his 70s presented for referral with a CW tumor in the left anterior chest. The tumor was located in the left anterior CW and was suspected to have pulmonary, thymic, and pectoralis major and minor invasions. Surgical resection included removal of the 2nd through the 4th ribs, partial left upper lobectomy, and partial thymectomy. The large CW defect was reconstructed via rigid reconstruction using a titanium mesh plate with a dual-mesh ePTFE sheet sewn inside. Pathological examination revealed a sarcomatoid malignant pleural mesothelioma. Three years after surgery, the patient remained recurrence-free. Despite radiographic evidence of titanium plate cracking, the pulmonary function and thoracic mechanics were preserved.

**CONCLUSIONS:**

The combination of ePTFE sheets, which have a smooth surface to protect the lungs and excellent tissue affinity, and titanium mesh plates, which are sufficiently rigid to maintain thoracic function, is an excellent method of rigid reconstruction that takes advantage of the strengths of each material. This technique is feasible and versatile and ensures short-term postoperative stability. Nevertheless, the long-term safety and potential complications warrant further clinical evaluation.

## Abbreviations

CW
: chest wall
ePTFE
: expanded polytetrafluoroethylene

## INTRODUCTION

Thoracic surgeons frequently manage neoplastic, traumatic, and congenital conditions that compromise the CW and require resection and reconstruction.^1)^ Maintaining CW integrity is essential for protecting intrathoracic organs and preserving respiratory mechanics. Wide excision with tumor-free margins is recommended, particularly for malignant tumors.^2)^ Deep lesions involving bone or soft tissue often result in rib or sternal defects, requiring rigid reconstruction.^3)^ The primary goals of reconstruction are to prevent paradoxical CW motion, preserve respiratory function, and protect the vital organs. Secondary aims include preventing scapular depression and maintaining cosmetic appearance.^4)^ However, indications for reconstruction remain controversial and are often based on the surgeon’s clinical judgment.^5,6)^

Titanium is widely used for CW reconstruction owing to its excellent biocompatibility, corrosion resistance, and favorable strength-to-weight ratio compared to steel.^7,8)^ Mesh-type titanium implants are particularly advantageous for conforming to the thoracic curvature. When combined with dual-surface ePTFE sheets—featuring a smooth side to reduce adhesion and a textured side to promote tissue integration—this technique enhances both surgical handling and safety by protecting the lungs and sutures.

## CASE PRESENTATION

**Fig. 1:**
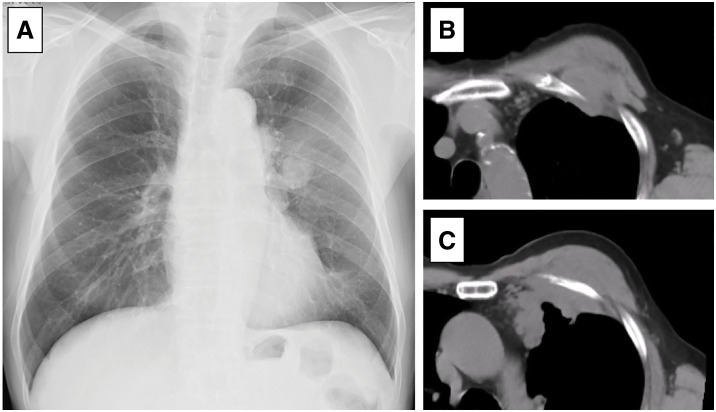
Preoperative chest imaging. (**A**) Chest radiograph showing a tumor in the left thoracic region. (**B**, **C**) CT showing a chest wall tumor suspected to involve the left upper lobe, thymus, and pectoralis major and minor muscles.

**Fig. 2:**
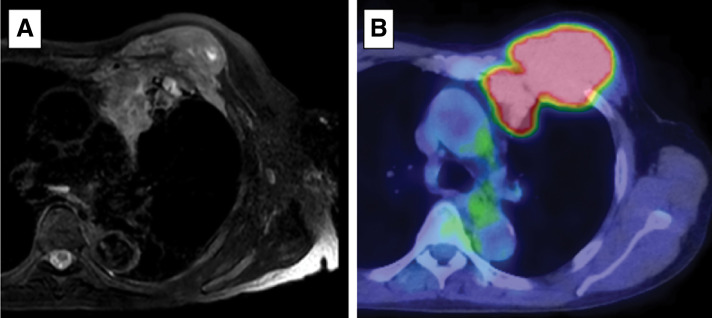
Preoperative MRI and FDG-PET. (**A**) Axial MRI section of a fat-suppressed T2-weighted sequence showing the left CW and an anterior mildly hyperintense signal. (**B**) Axial FDG-PET section overlaid on the CT scan showing the left CW tumor’s high glucose uptake, with a maximum standardized uptake value of 16.0. CW, chest wall; FDG, fluorodeoxyglucose

A man in his 70s was referred to our hospital for the evaluation of a CW tumor located in the left anterior thorax. Chest radiography and CT revealed a mass in the left anterior CW with suspected invasion of the left upper lobe of the lung, thymus, and pectoralis minor and major muscles (**Fig. 1**). MRI revealed an isointense lesion on T2-weighted images and a mildly hyperintense signal on fat-suppressed T2-weighted sequences (**Fig. 2A**). On fluorodeoxyglucose PET, the tumor exhibited high glucose uptake with a maximum standardized uptake value of 16.0 (**Fig. 2B**).

**Fig. 3:**
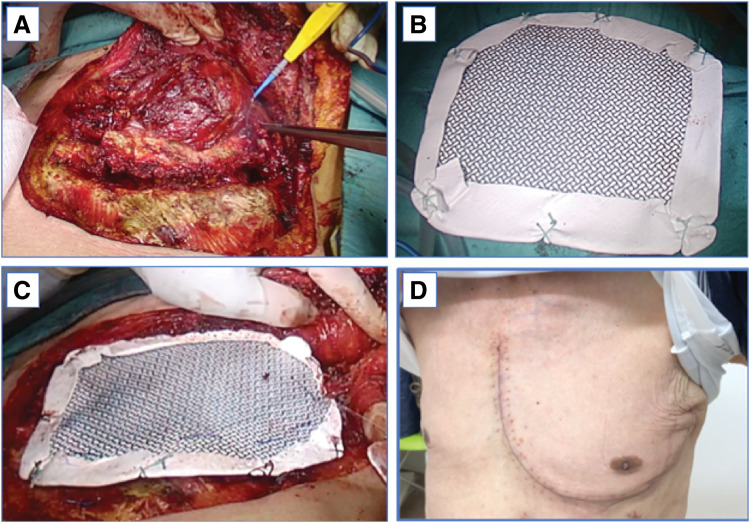
Intraoperative view of chest wall resection and reconstruction. (The **Supplementary Video** shows the reconstruction procedure in detail.) (**A**) Left chest wall with an L-shaped skin incision. The skin is retracted, revealing the tumor. (**B**) Preparation of the reconstruction material: A dual-surface ePTFE sheet was sewn onto the inner (lung-facing) side of a titanium mesh plate. (**C**) Application to the chest wall defect: The reconstruction material was placed on the outside of the ribs and fixed with wires and sutures. (**D**) Chest appearance 1 month after surgery: The chest wall was well reconstructed with no respiratory failure. ePTFE, expanded polytetrafluoroethylene

Percutaneous needle biopsy suggested a malignant pleural tumor, and the patient was scheduled for surgical resection. The resection line was carefully planned with CT and MRI imaging preoperatively and finalized intraoperatively. Through a left-sided L-shaped skin incision, en bloc resection of the CW, including the 2nd through 4th ribs and segments of the pectoralis major and minor muscles, was performed, along with partial resection of the left upper lobe and partial thymectomy. We secured a 2.5 cm margin on the sternal side without sternotomy to avoid excessive invasiveness, a 3–4 cm margin dorsally, preserved the 1st rib cranially with 2 cm margin, and preserved the 5th rib caudally with a 2 cm margin. To secure deep margins, we planned and performed layered resection of the pectoral muscles and partial resection of adjacent structures (lung and thymus). The resulting large CW defect, measuring approximately 12 × 12 cm, was reconstructed using a rigid titanium mesh plate (0.4 mm thick; 20 × 20 cm; MatrixNEURO Contourable Mesh; DePuy Synthes, Raynham, MA, USA), which was trimmed to approximately 16 × 14 cm. A dual-surface ePTFE sheet (18 × 24 cm; GORE DUALMESH; W. L. Gore & Associates, Flagstaff, AZ, USA) had been pre-sewn onto the lung-facing surface of the mesh to reduce adhesion and enhance tissue compatibility (**Fig. 3** and **Supplementary Video**).

**Fig. 4:**
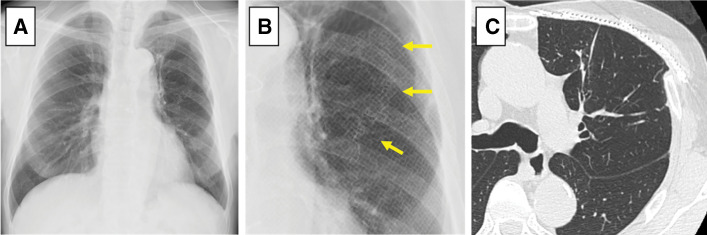
Radiological findings 3 years after surgery. (**A**) Chest radiograph showing good lung inflation. (**B**) Zoomed chest radiograph showing a crack in the titanium mesh plate (yellow arrows). (**C**) CT showing no significant lung damage, and that the reconstructed material is well-aligned.

Histopathological examination confirmed a sarcomatoid malignant pleural mesothelioma with negative surgical margins. The patient has remained recurrence-free for 3 years postoperatively. Although a crack in the titanium plate was observed (**Fig. 4**) and paradoxical movement resembling flail chest was slightly noted, pulmonary function remained preserved, with a postoperative percent predicted forced vital capacity of 115.3% (preoperative: 129.4%; change: −14.1%). The left lung volume increased slightly to 2205 mL (preoperative: 2138 mL, +3.1%), indicating that acceptable thoracic function was maintained.

## DISCUSSION

The approach to CW reconstruction depends on several factors, including defect size, scapular involvement, protection of internal organs, and the risk of herniation.^9,10)^ Rigid reconstruction is typically indicated for defects exceeding 5 or 10 cm near the scapula.^3,11)^ Anterior and anterolateral defects are particularly unstable and often require structural support.^12)^ Although the resection of 3 or more ribs is often considered an indication for reconstruction,^13,14)^ standardized criteria are lacking.

Titanium remains the material of choice due to its superior mechanical strength, biocompatibility, and compatibility with imaging modalities such as CT and MRI.^7,8,15)^ Mesh-type implants offer better anatomical conformity and versatility in complex reconstructions.

In this case, an ePTFE sheet was sutured to the lung-facing surface of the titanium mesh to reduce direct adhesion between the implant and the lung.^4)^ ePTFE is a flexible, elastic, and microporous material with a low risk of infection, making it ideal for thoracic use.^16)^ The preapplication of the ePTFE sheet before placing titanium on the CW improves handling during surgery and acts as a protective layer to prevent damage to the sutures and wires caused by the sharp edges of the titanium.

Several retrospective studies from Italy and China have reported good short-term outcomes with titanium meshes, with minimal complications.^17,18)^ However, there are concerns regarding their long-term durability. Berthet et al. found a high failure rate in titanium bar implants, especially in the anterior-inferior position, possibly due to repeated stress during respiration.^19)^ Even mesh-type implants may fracture under certain conditions, particularly when thinner variants (e.g., 0.4 mm) are used.^18)^ In our case, a crack was observed on the cranial side of the plate, extending to approximately two-fifths of its length. Although the defect was not severe and the patient reported no symptoms such as dyspnea, paradoxical movement resembling flail chest was slightly noted during inspiration. Given the potential for progression over time, this finding warrants long-term surveillance.

For future improvements, the development of more durable materials and designs is essential. Possible strategies include increasing plate thickness beyond 0.5 mm, incorporating honeycomb or other structural reinforcements, using alloy materials, and avoiding the placement of fixation holes near central regions subjected to maximal bending stress. Furthermore, securing adequate fixation length is critical. Instead of trimming the plate to the minimum required size, careful selection and shaping to ensure sufficient fixation margins may represent an immediately applicable measure to reduce mechanical stress and enhance stability.

Respiratory function is a critical outcome metric in CW reconstruction. Multiple studies have reported postoperative declines in forced expiratory volume in 1 s and forced vital capacity, depending on the number of resected ribs and the reconstruction type.^13,20–22)^ Recently, CT-based volumetric analysis has been proposed to objectively assess changes in lung capacity.^23)^

In this case, despite the late-stage fracture of the titanium mesh, lung volume and respiratory function remained stable, suggesting that functional reconstruction was achieved. However, further long-term data are necessary to evaluate implant longevity and to improve material and design choices.

## CONCLUSIONS

The combination of titanium mesh plates and dual-surface ePTFE sheets provided a practical and effective method for rigid CW reconstruction. Titanium offers rigidity and anatomical conformity, whereas ePTFE protects against lung adhesion on the smooth side and promotes tissue integration on the textured side. This technique is simple, adaptable, and suitable for achieving short-term postoperative stability. Nevertheless, further long-term clinical studies are essential to evaluate the mechanical durability of this approach and to optimize future material designs and surgical strategies for CW reconstruction.

## SUPPLEMENTARY MATERIALS

Supplementary Video

## DECLARATIONS

### Availability of data and materials

Data sharing is not applicable to this article because datasets were neither generated nor analyzed for the case report.
